# Combined fractional CO2 laser with topical tioconazole versus Q-switched Nd-YAG laser in the treatment of onychomycosis; a randomized comparative trial

**DOI:** 10.1007/s10103-024-04214-9

**Published:** 2024-11-12

**Authors:** Khadiga Sayed, Manar Khaled, Abdallah Gad, Amira Elbendary

**Affiliations:** 1 Dermatology Department, Kasr alainy Faculty of Medicine, Cairo University, Cairo, Egypt; 2 Lowell General Hospital, Lowell, MA, USA; 3 Biostatistics and Cancer Epidemiology Department, National Cancer Institute, Cairo University, Giza, Egypt

**Keywords:** Onychomycosis, Fractional CO2 laser, Topical tioconazole, Q-switched nd: YAG 1064 nm, OSI score, KOH

## Abstract

Treatment of onychomycosis includes topical and systemic agents. However, prolonged use of oral treatment could cause adverse effects and topical antifungal agents have limited penetration. To compare the clinical efficacy and the safety of fractional CO_2_ laser combined with topical tioconazole nail solution versus Q-switched 1064 Nd: YAG laser in the treatment of fingernail onychomycosis. This randomized comparative clinical trial was conducted on 13 patients (47 nails) with fingernail onychomycosis. Patients were randomized to receive either fractional CO2 laser combined with topical tioconazole or Q-Switched Nd: YAG 1064 nm laser every 2 weeks for 3 months followed by a 1-month follow up assessment. Onychomycosis severity index (OSI) score, Dermatology life Quality Index (DLQI) score, patient satisfaction score, dermoscopic evaluation and KOH examination were used for assessment of improvement. OSI showed improvement after treatment in both arms (from 16.17 to 10.92 in fractional CO2 arm (***p* = 0.026)** and 23.13 to 22.43 (***p* = 0.92)).** When comparing both groups OSI score significantly reduced in the fractional CO2 laser combined with tioconazole more than the Q-switched laser group (*p* = 0.002). The mean DLQI score significantly improved in both groups but no statistically significant difference between the two groups. Significant improvement in patient satisfaction score was noted in both groups. Mycological cure using KOH examination was detected in both groups (44.4% in the CO2 group and 56.5% in the Q-switched group) with no significant difference **(P value < 0.05).** Ruin pattern keratosis found to be the most dermoscopic pattern to be associated with poor OSI score improvement. Fractional CO2 laser combined with topical tioconazole is more efficient in treatment of onychomycosis than Q-Switched Nd: YAG 1064 nm laser group as regards clinical improvement but both have comparable effect on mycological cure. Their use as adjuvant treatment rather than alone is recommended to ensure mycological cure in onychomycosis.

## Background

Onychomycosis (OM) is a fungal infection of the nail, with an estimated global prevalence of 5.5%. The disease can affect quality of life by causing discomfort and social limitations [1].

The recommended treatment regimen for OM includes topical and oral treatment regimens or a combination of both. However, topical agents show limited nail permeation and oral treatment may be associated with systemic side effects due to the requirement of prolonged intake duration to reach therapeutic effect [2].

Tioconazole is a synthetic imidazole antimicrobial agent used for the treatment of nail infections. A 28% nail solution has been formulated and it should be applied twice daily for prolonged periods usually of at least 6 months. It is generally well tolerated by patients. Local reactions including redness, burning, and itching have been the only adverse experiences that have been documented [3].

The mechanism of action for the fractional CO2 laser on nails involves a photothermal effect, with eventual micro-explosion, resulting in catabolism [4]. The direct killing of the fungus is also facilitated by elevated local temperatures, as fungi are susceptible to thermal damage at temperatures exceeding 40 °C, causing protein denaturation and deactivation [5]. Furthermore, the fractional CO2 laser may contribute to inhibiting fungal growth by causing vaporization and exfoliation of the local tissue around the affected nail. This process leads to diffuse remodeling and simultaneously destroys the fungal growth environment [6]. Additionally, the fractional CO2 laser generates micro-holes in the nail plate, enhancing the penetration of topical antifungal medications into the nail bed [4, 7].

The impact of the Q-switched Nd-YAG laseris suggested to result from non-specific tissue heating, leading to increased circulation through vasodilatation and stimulation of the immune system [8]. Galvan Garcia and colleagues (2014) argue that the Q-switched Nd: YAG laser generates high-energy peaks with numerous repetitions, causing minimal tissue warming and producing impact energy that mechanically damages only the fungi. They propose that the mechanism underlying laser therapy is selective photothermolysis, dependent on pigment type, light characteristics, and pulse frequency [9].

In addition, a study notes that the 532-nm Q-switched Nd: YAG laser can inhibit Trichophyton rubrum due to its significant content of the endogenous pigment xanthomegnin, which absorbs light between 406 and 555 nm. On the other hand, the 1064-nm wavelength of the Q-switched Nd: YAG laser, beyond the absorption spectra of xanthomegnin, also exhibits inhibitory effects on T. rubrum. This inhibitory impact is attributed to the abundance of melanin in the cell wall of Trichophyton species [5, 10].

The aim of this study is to compare the clinical efficacy and the safety of fractional CO_2_ laser combined with topical tioconazole nail solution versus Q-switched 1064 Nd: YAG laser in the treatment of fingernail onychomycosis.

## Materials and methods

This study was designed as a prospective randomized comparative trial (RCT). It was conducted at the Dermatology Outpatient Clinic, Dermatology Department, Faculty of Medicine, Cairo University (Kasr Al Aini hospital). Patients were recruited from April 2022 to December 2022. The study design was approved by both the Department Scientific and Ethical Committee Boards. To comply with the rules of conducting clinical trials, the study was published at the website of www.clinicaltrials.gov under the number NCT05999474.

13 patients with 47 fingernail onychomycosis presenting consecutively to the Dermatology Outpatient Clinic were assessed for eligibility for the therapeutic phase of the study. Diagnosis was ensured to be documented clinically, microscopically and dermoscopically. The inclusion criteria were patients of both genders aged > 18 years, with any type of finger onychomycosis. Any pregnant patient or patients having localized bacterial infection around the affected nail or concomitant nail disease that causes nail dystrophy or discoloration such as psoriasis, lichen planus and atopic dermatitis that may interfere with diagnosis and treatment, or patients with history of intake of oral antifungal medication within the last 3 months or the use of topical antifungal medication within the last 2 weeks prior to the laser treatment were excluded from the study.

After signing the consent, each patient was subjected to detailed history regarding the onset, course, duration of onychomycosis, exacerbating factors as previous trauma, nail polish and continuous contact with water as well as any past medical problems.

Nail culture was not chosen as method for diagnosis and assessment due to its difficulty to isolate the fungal elements from the contaminants, high percentage of false negative results reaching up to 30%, prolonged time until results, its expense, and presence of dermoscopic evaluation which was reported in literature to have comparable results in diagnosing onychomycosis **(Nada EEA**,** El Taieb MA**,** El-Feky MA**,** et al. Diagnosis of onychomycosis clinically by nail dermoscopy versus microbiological diagnosis.*Arch Dermatol Res***. **2020;312** (3):**207–212. doi**:10.1007/s00403-019-02008-6**).**

The nail plate and surrounding soft tissue were cleaned with 70% ethyl alcohol to prevent contamination prior to sample collection. Adequate nail clippings or scrapings from subungual hyperkeratosis were collected in a sterile container from each patient using a sterile nail clipper or blade. The collected samples were subjected to KOH examination on glass slides using potassium hydroxide (20%) in a solvent of 60% water and 40% dimethyl sulfoxide (DMSO) to hasten keratin dissolution. They were then left for 10 min and heated for 15 s. Subsequently, they were subjected to light microscopy examination to determine the presence or absence of fungal elements (both yeasts and dermatophytes).

Patients were randomly allocated into either one of the arms of the trial, the arm 1 (Fractional CO2 combined with tioconazole) or the arm 2 (Q-switched Nd-YAG). A 1:1 assignment was adopted. Accordingly, arm 1 contained 23 nails and arm 2 contained 24 nails. The results were assessed by blinded investigator.

**Fig. 1:**
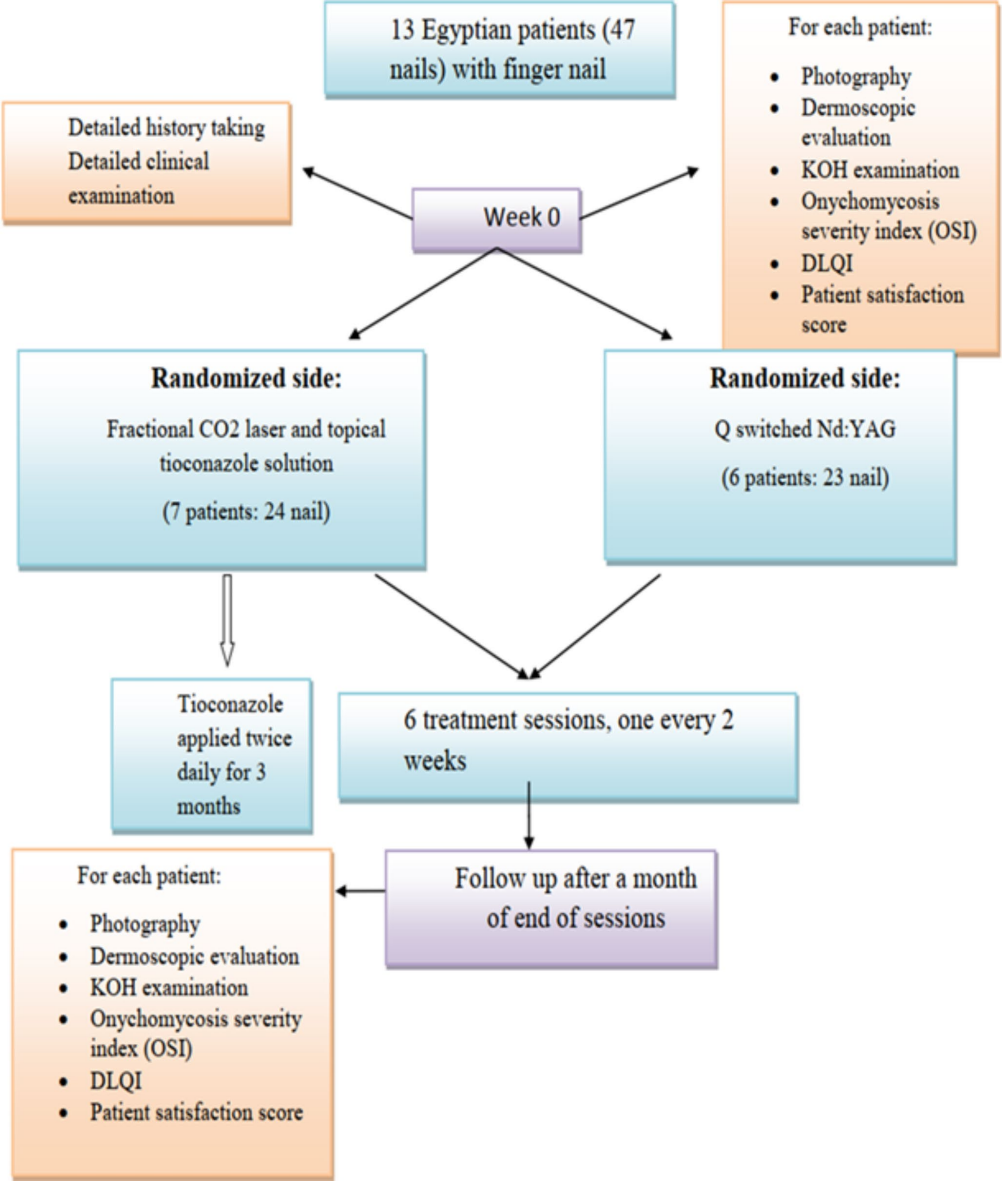
Patient flow chart demonstrating the sequence of the study according to CONSORT guidelines for reporting randomized controlled trials

Recruited patients received 6 sessions of either fractional CO2 laser combined with topical tioconazole or Q-Switched Nd: YAG 1064 nm laser with an interval of 2 weeks for 3 months. Topical anaesthetics (lidocaine and pridocaine 5%) were applied for 60 min under occlusion before laser sessions (Fig. 1).

Fractional $\:{CO}_{2\:}$laser (10.600 nm wavelength) (DEKA Smartxide DOT) was used in the current study. Sessions were done every 2 weeks for 3 months (6 sessions) with the following parameters: power 12 watts, pulse duration of 500µs, spacing of 700 μm and stack 2 or 3 according to nail thickness. In one session two passes across each nail plate were performed. Topical tioconazole nail solution was applied in between the sessions twice daily. Each nail was fully covered with the laser beam, including the areas of the hyponychium and the proximal and lateral nail folds.

Sessions using Qs-Nd: YAG (1064 nm) laser Fotona’s $\:{\text{Q}\text{X}\:\text{M}\text{A}\text{X}}^{\text{\circledR\:}}$. were done every 2 weeks for 3 months (6 sessions) with the following parameters: 3 mm spot size and a power level of 4 which delivers 14 joules/cm2, 9 billionths of a second pulse duration, and a 2.5 Hz frequency. In one session two passes across each nail plate were performed. Each nail was fully covered with the laser beam, including the areas of the hyponychium and the proximal and lateral nail folds.

### Patients’ assessment

Assessment done by one blinded investigator at day 0 and at end of study (EOS) using KOH examination and dermoscopic evaluation [11].

Onychomycosis Severity Index (OSI) score was assessed for each patient. Points were given based on area percentage of nail involvement (0–5points), proximity of disease to matrix (1–4 points, with 5 points if the matrix is involved) and presence (10 points) or absence (0 points) of dermatophytoma or subungual hyperkeratosis > 2 mm. The score for the area of involvement (range, 0–5) was multiplied by the score for the proximity of disease to the matrix (range, 1–5), and 10 points were added if a longitudinal streak or a patch (dermatophytoma) was present or if there was greater than 2 mm of subungual hyperkeratosis. It classifies nail involvement as mild (1- points), moderate (6–15 points) or severe (16–35 points) Table 1 [12].

**Table 1:** Onychomycosis severity index

Area of involvement		Proximity of onychomycosis to nail matrix		Presence of dermatophytoma or subungual hyperkeratosis > 2 mm	
Percentage of affection	Number of points	Area of involvement from distal edge	Number of points	Presence	Number of points
1–10	1	< 25%	1	Yes	10
11–25	2	25-50%	2	No	0
26–50	3	> 50-75%	3		
51–75	4	> 75%	4		
76–100	5	Matrix involvement	5		

This score is adopted from Carney et al., 2011

Dermatology life quality index (DLQI) questionnaire was filled by each patient before starting the laser sessions and four weeks after the last session. The responses were scored from 0 to 3 (0 being the minimum, 3 being the maximum) [13].

Patient satisfaction score, which is a score given to assess the extent of satisfaction was filled by each patient before starting the study and four weeks after the last session. The patient chooses a four-scale grading: Poor, fair, good and excellent (Table 2) [14].

**Table 2:** Patient satisfaction score

Before treatment	Poor; response rate: (0-25%)	□
	Fair; response rate: (25-50%)	□
	Good; response rate: (50-75%)	□
	Excellent; response rate: (75-100%)	□
4 weeks after end of treatment	Poor; response rate: (0-25%)	□
	Fair; response rate: (25-50%)	□
	Good; response rate: (50-75%)	□
	Excellent; response rate: (75-100%)	□

## Patient evaluation at EOS

At the end of the study (3 months), therapeutic efficacy assessed 1 month after end of the study for clinical response.

Excellent response is defined as a global improvement of the treated nail of > 90% according to OSI score.

Very good response is defined as improvement of 75–90%.

Good response is defined as improvement of 50–75%.

Poor response is defined as improvement of < 50%.

No response Defined as no change in OSI score.

Relapse is defined as recurrence of nail lesions after it had improved or resolved during the followup period which is one month.

Overall good responses: 50% reduction or more in OSI score.

Overall poor responses: < 50% reduction in OSI score or no response or relapse.

Besides that, dermoscopic and microscopic evaluation using KOH to evaluate mycological cure were done.

## Statistical method

Data analysis was performed using IBM SPSS Statistics for Windows, version 23 (IBM Corp., Armonk, N.Y., USA). Categorical data were expressed as percentages and numerical data were summarized into means. Comparisons between categorical data were conducted using Pearson’s Chi-square (χ2) test or Fisher’s exact test when appropriate. Comparisons between numerical data were conducted using the Mann-Whitney test. All tests were two-tailed and p-values of less than 0.05 were considered statistically significant.

## Results

The current study included 13 onychomycosis patients, 3 males (23%) and 10 females (76.9%), their ages ranged from 19 to 72 years (mean 41.6 years). The number of included affected nails was 47, the mean duration of onychomycosis was 20.8 months. 28 (59.5%) nails were distal lateral subungual onychomycosis (DLSO) and 19 (40.4%) nails were total dystrophic onychomycosis (TDO). Comparing both groups of the study as regards baseline characteristics revealed significant difference in the mean OSI score being higher in the Q-switched Nd: YAG group **(*P*** **= 0.019).** Affected nails and different factors that could affect occurrence of onychomycosis are summarized in Table 3.

**Table 3:** Patients’ basic characteristics of the studied nails

Variables		*N* = 47 Number (%)
Predisposing factors	Excessive contact with water	41 (87.2%)
	Previous nail trauma	7 (14.8%)
	Nail polish use	12 (25.5%)
	Diabetes mellitus	2 (4.2%)
Complication of onychomycosis	Recurrent paronychia	22 (46.8%)
	Cellulitis	5 (10.6%)
History of previous treatment	Topical treatment	26 (55.3%)
	Systemic treatment	17 (36.1%)
	Laser treatment	0
	Surgical treatment	5 (10.6%)

Dermoscopic examination at start of the study showed that longitudinal striae pattern (93.6%), spiked pattern (89.4%) and distal irregular pattern (70.2%) are the commonest dermoscopic patterns found.

## Clinical assessment

### **Efficacy of fractional CO2 laser combined with topical tioconazole**

**Fig. 2:**
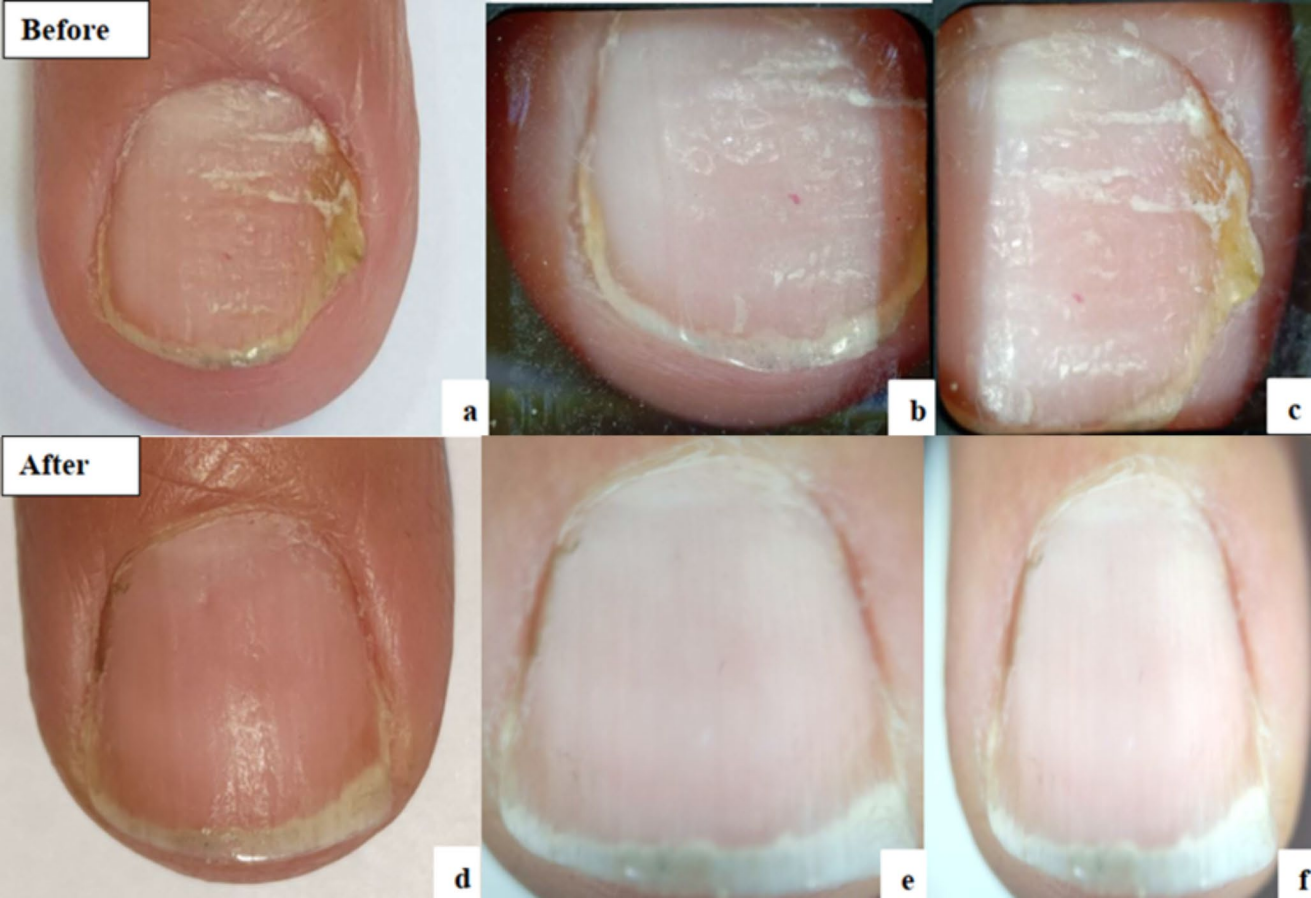
Fractional CO2 group: **a**: Clinical photo before treatment. **b**&**c**: Dermoscopic photos before treatment. **d**: Clinical photo after treatment. **e**&**f**: Dermoscopic photos after treatment

**Fig. 3:**
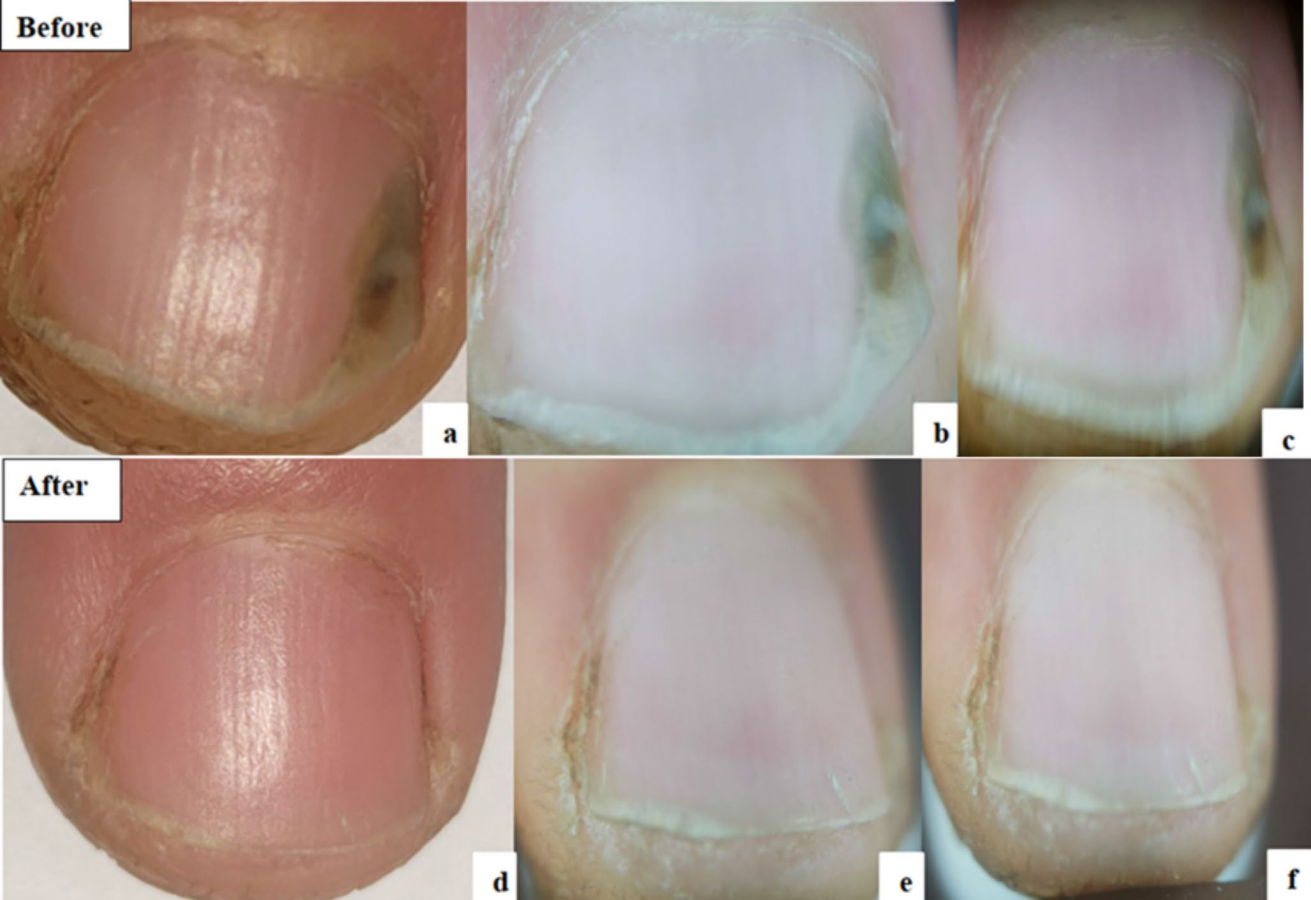
Fractional CO2 group: **a**: Clinical photo before treatment. **b**&**c**: Dermoscopic photos before treatment. **d**: Clinical photo after treatment. **e**&**f**: Dermoscopic photos after treatment

The mean OSI Score before starting treatment in the nails treated with fractional CO2 laser was 16.17 which was significantly decreased with a mean of 10.92 after treatment (***P*** **= 0.026)**. Detailed results of the components of OSI showed significant improvement in area of involvement from a mean of 3.71 to 2.75 **(*P*** **= 0.006)** and significant improvement in its percentage from mean of 54.79–38.92% **(*P*** **= 0.017)**. The amount of involvement from the distal edge was also markedly improved. No significant change noted regarding the mean score of presence of dermatophytoma or subungual hyperkeratosis. Significant improvement was also found in DLQI and satisfaction of the patients ***(P*** **< 0.001)** (Table 4) (Figs. 2 and 3).

**Table 4:** DLQI, satisfaction score and OSI scores before and after treatment in the fractional CO2 group

Variables		Before (mean)	After (mean)	*P*-value
DLQI		3	1.58	< 0.001*
Satisfaction score		0.08	1.79	< 0.001*
Mean OSI score		16.17	10.92	0.026*
Components of OSI score	Area of involvement	3.71	2.75	0.006*
	Percentage of area involvement	54.79	38.92	0.017*
	Amount of involvement from distal edge	3.5	2.83	0.068
	Presence of dermatophyoma or subungual hyperkeratosis	6 (25%)	4 (16.7%)	0.477

*P value < 0.05 is considered significant

Detailed clinical evaluation of the different responses of the studied fractional CO2 group demonstrated that 2 (8.3%) nails showed excellent response, 2 (8.3%) nails showed very good response, 5 (20.8%) nails showed good response. 6 (25%) nails showed poor response, 8 (33.3%) nails showed no response and 1 (4.1%) nail relapsed.

### Efficacy of Q-switched nd: YAG laser before and after treatment

**Fig. 4:**
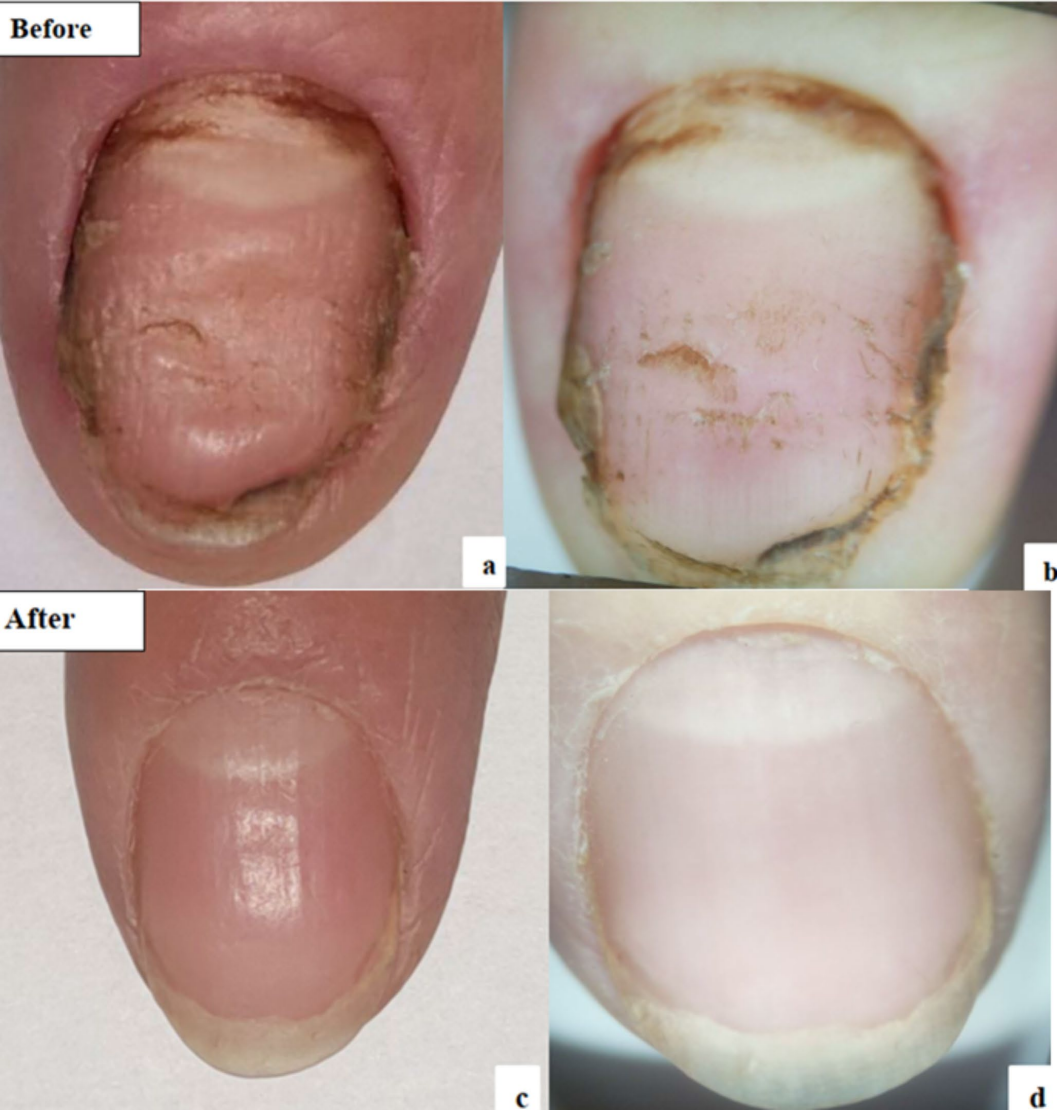
Q-switched Nd-YAG group: **a**: Clinical photo before treatment. **b**: Dermoscopic photo before treatment. **c**: Clinical photo after treatment. **d**: Dermoscopic photo after treatment

**Fig. 5:**
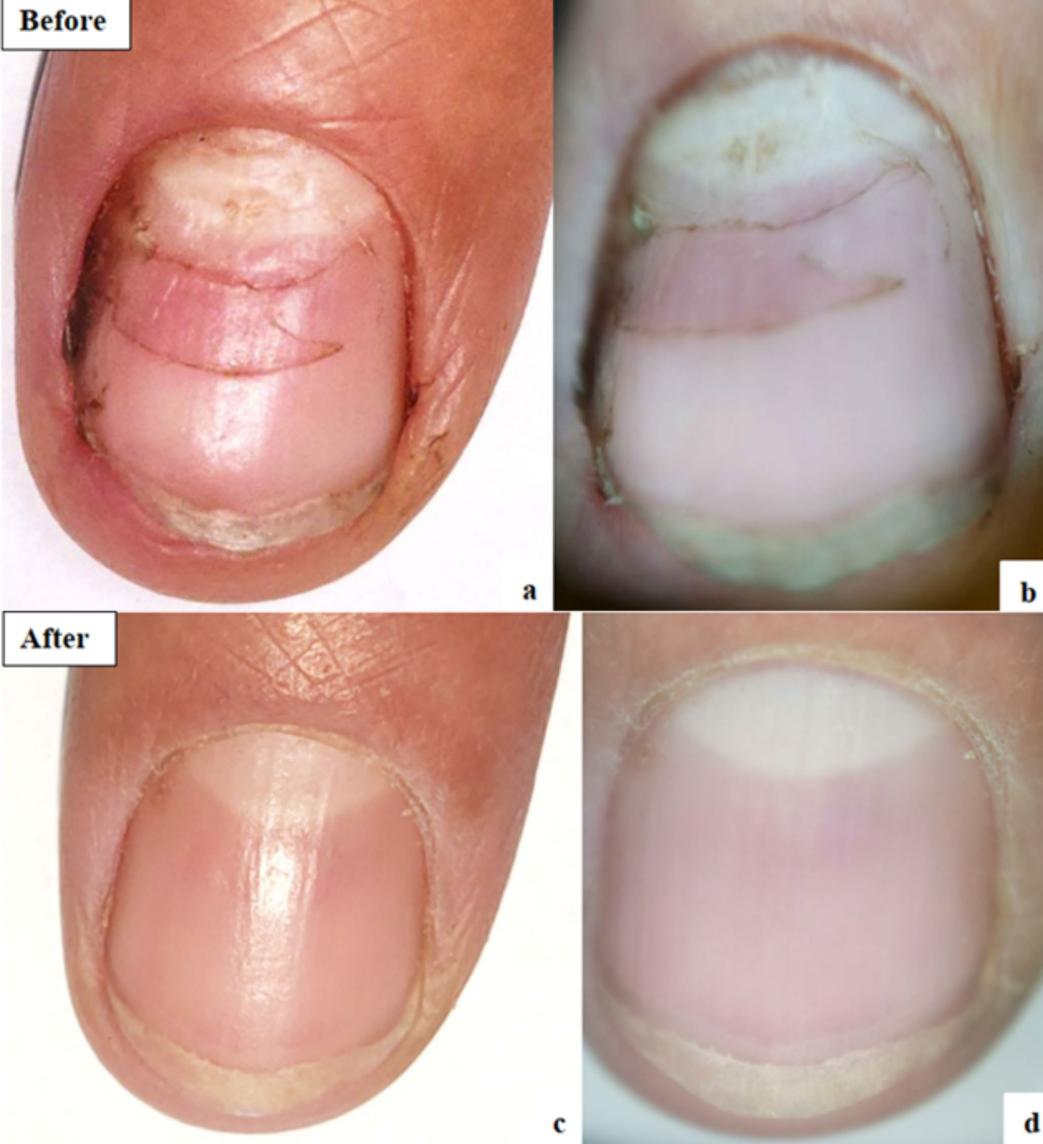
Q-switched Nd-YAG group: **a**: Clinical photo before treatment. **b**: Dermoscopic photo before treatment. **c**: Clinical photo after treatment. **d**: Dermoscopic photo after treatment

The mean OSI Score before starting treatment in the nails treated with Q- switched laser was 23.13 which was decreased slightly to a mean of 22.43 after treatment. Detailed results of the components of OSI also showed no significant improvement after treatment **(*P*** **> 0.05)**. Significant improvement was found in DLQI and satisfaction of the patients **(*P*** **< 0.05)** (Table 5; Figs. 4 and 5).

**Table 5:** DLQI, satisfaction score and OSI scores before and after treatment in Q-switched nd: YAG group

Variables		Before (mean)	After (mean)	*P*-value
DLQI		2.43	1.78	0.026*
Satisfaction score		0.87	1.52	< 0.001*
Mean OSI score		23.13	22.43	0.92
Components of OSI score	Area of involvement	4.17	3.83	0.46
	Percentage of area involvement	71.74	64.21	0.494
	Amount of involvement from distal edge	4.17	3.78	0.369
	Presence of dermatophyoma or subungual hyperkeratosis (Number%)	12 (52.2%)	15 (65.2%)	0.37

*P value < 0.05 is considered significant

Detailed clinical evaluation of the different responses of the studied Q-switched Nd: YAG group demonstrated that 1 (4.3%) nails showed excellent response, 1 (4.3%) nails showed very good response, 1 (4.3%) nails showed good response, 5 (21.7%) nails showed poor response, 9 (39.1%) nails showed no response and 6 (26%) nails relapsed.

### Comparison of scores between both groups after treatment

The mean onychomycosis severity index (OSI) showed statistically significant difference between both groups after treatment with more improvement in the fractional CO2 group **(*P*value < 0.05)** (Table 6). As regards DLQI score, no significant difference between both groups after treatment, and as regards satisfaction score, it showed statistically significant difference between both groups after treatment with more improvement in the fractional CO2 group **(*P*value < 0.05)**.

**Table 6:** Comparison of scores between both groups after treatment

Variables		Fractional CO2group (Mean)	Q-switched Nd: YAG group (Mean)	*P*-value
DLQI		1.58	1.78	0.769
Satisfaction score		1.79	1.52	0.049*
Mean OSI		10.92	22.43	0.002*
Components of OSI score	Area of involvement	2.75	3.83	0.004*
	Percentage of area of involvement	38.92	64.21	0.005*
	Amount of involvement from distal edge	2.83	3.78	0.011*
	Presence of dermatophytoma or subungual hyperkeratosis (N%)	4 (16.7%)	15 (65.2%)	< 0.001*

### Changes in dermoscopic features in both groups after treatment

**Fig. 6:**
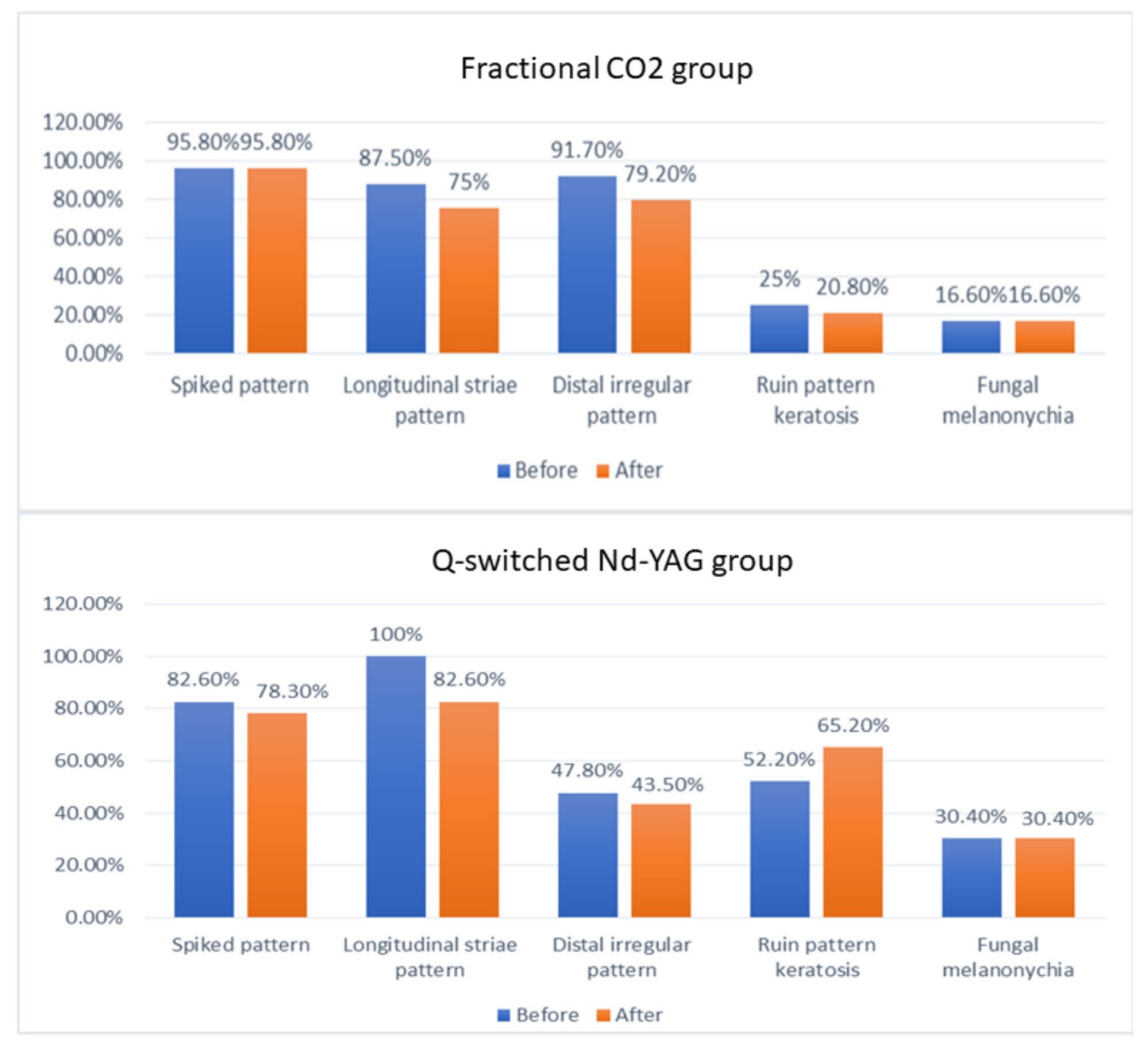
Bar chart illustrating the percentages of different dermoscopic features seen in onychomycosis and their changes before and after treatment in both laser groups

Regarding the disappearance of the different dermoscopic patterns after treatment in the nails of the fractional CO2 group, some nails showed disappearance of the longitudinal striae pattern, distal irregular pattern and ruin pattern keratosis after treatment but that was statistically insignificant **(*P* value > 0.05)**. No nails showed disappearance of the spiked pattern and the fungal melanonychia pattern as shown in (Fig. 6). While in the Q- switched Nd: YAG group, some nails showed disappearance of the spiked, longitudinal striae pattern and distal irregular pattern but that was statistically insignificant **(*P* value > 0.05)**. No nails showed disappearance of the fungal melanonychia pattern.

### Changes in KOH examination in both groups before and after treatment

The number of nails in fractional CO2 group that showed positive KOH examination decreased significantly after treatment in the fractional CO2 group (from 18 to 8) **(*P* value = 0.004)** and this was also present in Q-switched ND-YAG laser. from 23 nails to 13 nails **(*P* value < 0.001).** However, no statistically significant difference in percentage of nails that turned KOH negative after treatment between both groups **(*P* value > 0.05).**

Regarding paronychia, there was marked improvement after treatment in both treatment groups; in the fractional CO2 group, 41.7% of nails had paronychia before treatment turned 0% after treatment which was statistically significant and in the Q- switched Nd: YAG group, 13% of nails had paronychia before treatment also turned 0% after treatment but this was statistically insignificant.

### Side effects after treatment

Four (16.6%) nails in the fractional CO2 laser group showed persistent pixel stamp marks, six nails (25%) showed minute haemorrhage and the other fourteen nails (58.3%) showed no side effects. Three (13%) nails in the Q-switched Nd: YAG group showed minute haemorrhage and the other twenty (86.9%) nails showed no side effects.

## Discussion

The FDA has approved numerous laser treatments for a “temporary increase in clear nail in patients with onychomycosis,” but lasers have not been established as a cure, and there is a scarcity of peer-reviewed literature addressing the subject [15]. Among these FDA-approved lasers for onychomycosis treatment are Fractional CO2 laser and Q-switched laser [5].

Our study aimed to compare the clinical efficacy and safety of fractional CO2 laser combined with topical tioconazole nail solution versus Q-switched 1064 Nd: YAG laser in the treatment of fingernail onychomycosis. To the best of our knowledge, this study is the first to make such a comparison in the treatment of onychomycosis.

In the current study, it was observed that distal and lateral subungual onychomycosis (DLSO) (59.6% of included nails) was the most predominant clinical type of onychomycosis, followed by TDO (40.4%). This aligns with other studies, which showed DLSO (62.5%) as the most prevalent, followed by (20.8%) with PSO and (12.5%) with TDO [16, 17].

Regarding therapeutic efficacy, a significant reduction in the mean OSI score was noted in nails treated with fractional CO2 laser, while there was no significant reduction in the mean OSI score in nails treated with Q-switched Nd: YAG laser. Overall, 37.5% of nails showed good responses in the fractional CO2 group, compared to only 13% in the Q-switched Nd: YAG group.

Our results align with studies reported in the literature. El-Tatawy and colleagues (2019) investigated the role of fractional CO2 laser and topical tioconazole 28% nail lacquer in the treatment of fingernail onychomycosis, finding significantly better improvement in both laser and combined groups compared to the topical group [18]. Similarly, Abd El-Aal and colleagues (2019) evaluated the efficacy of fractional carbon dioxide laser-assisted delivery of topical tazarotene versus topical tioconazole, showing comparable clinical responses in both groups [19].

In addition, Bhatta and colleagues (2016) studied the efficacy of fractional CO2 laser combined with a topical terbinafine cream, with 73.32% of patients having fully or more than 60% normal-appearing nails after 3 months from the last treatment [4].

In contrast to our study, Elmorsy and colleagues (2020) investigated the efficacy of Long Pulsed Nd: YAG (1,064 nm) Laser versus Q-Switched Nd: YAG (1,064 nm) Laser for the treatment of onychomycosis, with both groups showing statistically significant improvement in proximal nail plate measurements. However, clinical success was higher in group II at both the end of treatment and 6 months follow-up [20].

Galvan Garcia (2014) evaluated the efficacy of the 1064-nm Q-switched Nd: YAG laser in the treatment of onychomycosis, reporting a 93% clinical response rate within 3 months of the initial laser treatment and a 100% clinical response rate at 6 months [9].

The higher clinical efficacy of Q-switched Nd: YAG laser in other studies compared to our study could be explained by longer follow-up periods. In our study, the poorer results of Q-switched Nd-YAG laser compared to fractional CO2 laser may be due to a significantly higher baseline mean OSI for included nails in the Q-switched Nd: YAG group, and severe onychomycosis was associated with poor results in both treatment groups.

However, fractional CO2 laser was slightly better in improving severe onychomycosis. Although this was statistically insignificant, 3/11 nails improved versus 0/16 in the Q-switched Nd: YAG laser group. Although both treatment groups showed a statistically significant improvement in mean DLQI score, there was no statistically significant difference between them.

A statistically significant incidence of mycological cure in KOH examination was detected in both the fractional CO2 group and the Q-switched Nd: YAG group, with no significant difference between them. This is consistent with studies by Abd El-Aal and colleagues (2019) and El-Tatawy and colleagues (2019), where mycological cure rates were reported [18, 19].

However, the overall good responses associated with negative KOH microscopy after treatment were lower in the fractional CO2 group (33.3%) compared to the Q-switched Nd: YAG group (66.6%). Poor responses associated with negative KOH microscopy after treatment may be due to the possibility of false-negative results of KOH, with reported sensitivity ranging from 48 to 60% [21].

Regarding the disappearance of different dermoscopic patterns in the treated nails, both types of laser had comparable effects. In conclusion, Fractional CO2 laser combined with topical tioconazole is more efficient in the treatment of onychomycosis than Q-Switched Nd: YAG 1064 nm laser group in terms of clinical improvement, but both have a comparable effect on mycological cure. Both treatments are safe and are better used as adjuvant treatments rather than alone to ensure mycological cure in onychomycosis.

Limitations of the current study include the short follow-up time of one month, preventing a conclusion about how long the laser effect would last or further improve the condition.
